# Dye-sensitized solar cells under ambient light powering machine learning: towards autonomous smart sensors for the internet of things†

**DOI:** 10.1039/c9sc06145b

**Published:** 2020-02-13

**Authors:** Hannes Michaels, Michael Rinderle, Richard Freitag, Iacopo Benesperi, Tomas Edvinsson, Richard Socher, Alessio Gagliardi, Marina Freitag

**Affiliations:** Department of Chemistry, Ångström Laboratory, Uppsala University P. O. Box 523 SE-75120 Uppsala Sweden; Department of Electrical and Computer Engineering, Technical University of Munich Karlstraße 45 80333 Munich Germany; IT-Division, Uppsala University Dag Hammarskjölds Väg 7, P. O. Box 256 SE-75105 Uppsala Sweden; Department of Solid-state Physics, Ångström Laboratory, Uppsala University P. O. Box 534 SE-75121 Uppsala Sweden; Salesforce Research 172 University Avenue Palo Alto CA 94301 USA; School of Natural and Environmental Science, Bedson Building, Newcastle University NE1 7RU Newcastle upon Tyne UK marina.freitag@newcastle.ac.uk

## Abstract

The field of photovoltaics gives the opportunity to make our buildings ‘‘smart’’ and our portable devices “independent”, provided effective energy sources can be developed for use in ambient indoor conditions. To address this important issue, ambient light photovoltaic cells were developed to power autonomous Internet of Things (IoT) devices, capable of machine learning, allowing the on-device implementation of artificial intelligence. Through a novel co-sensitization strategy, we tailored dye-sensitized photovoltaic cells based on a copper(ii/i) electrolyte for the generation of power under ambient lighting with an unprecedented conversion efficiency (34%, 103 μW cm^−2^ at 1000 lux; 32.7%, 50 μW cm^−2^ at 500 lux and 31.4%, 19 μW cm^−2^ at 200 lux from a fluorescent lamp). A small array of DSCs with a joint active area of 16 cm^2^ was then used to power machine learning on wireless nodes. The collection of 0.947 mJ or 2.72 × 10^15^ photons is needed to compute one inference of a pre-trained artificial neural network for MNIST image classification in the employed set up. The inference accuracy of the network exceeded 90% for standard test images and 80% using camera-acquired printed MNIST-digits. Quantization of the neural network significantly reduced memory requirements with a less than 0.1% loss in accuracy compared to a full-precision network, making machine learning inferences on low-power microcontrollers possible. 152 J or 4.41 × 10^20^ photons required for training and verification of an artificial neural network were harvested with 64 cm^2^ photovoltaic area in less than 24 hours under 1000 lux illumination. Ambient light harvesters provide a new generation of self-powered and “smart” IoT devices powered through an energy source that is largely untapped.

## Abstract 2

Indoor light harvesters enable machine learning on fully autonomous IoT devices at 2.72 × 10^15^ photons per inference.

## Graphical abstract

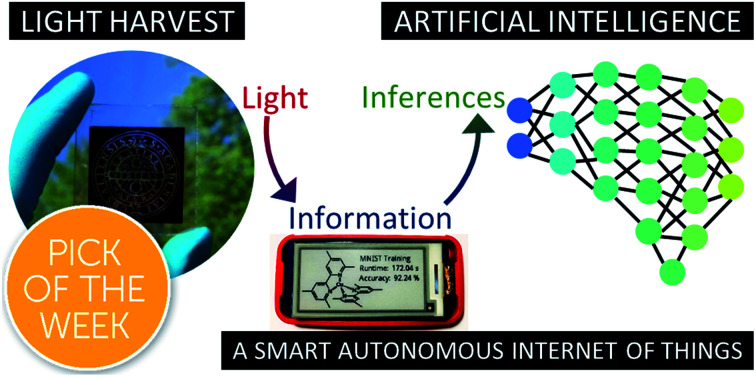

## Introduction

From conservation efforts and cleantech to tracking of environmental conditions and reductions in energy usage, every imaginable facet in the quest to reduce our carbon footprint is being explored anew through the IoT (Internet of Things). The IoT, as world-spanning networks of physical devices connected to the internet, marks an ever-growing field of technology. Such networks of autonomous smart sensing devices are poised to advance the exchange of information in smart homes, offices, cities, and factories.^1–4^ It is being argued that many aspects of our life will be mediated *via* 75 billion IoT devices by 2025, of which the majority will reside indoors. They will collect, communicate and process real-time data to optimize services and manufacturing processes, as well as to manage resources to reduce our energy consumption.^5,6^ Most importantly towards broad implementation, such IoT devices have to become autonomous, which requires a local power source with low or even zero maintenance. Therefore, it is crucial to find an energy source that yields high efficiencies in this environment.

In outdoor photovoltaics, a significant portion of the sun's spectrum is found in the red region of the visible light and at near-infrared wavelengths, which suits the strong spectral response of crystalline silicon or GaAs-based solar cells in this wavelength domain. On the contrary, the largest part of indoor illumination spectra, most commonly originating from fluorescent lamps, is found in the visible range between 400 and 650 nm. In this spectral region, diffuse ambient light provides universally available energy, which remains otherwise unused.^7–14^ Photovoltaic technologies based on amorphous silicon (a-Si),^15–17^ organic photovoltaics (OPV),^9,18,19^ and dye sensitized cells (DSC)^20–22^ have shown sufficient energy conversion in this region.

DSCs are well known for their high performance in ambient light. In 2017, Freitag *et al.* introduced a new dye-sensitized solar cell design with Cu^II/I^(tmby)_2_ (tmby = 4,4′,6,6′-tetramethyl-2,2′-bipyridine) as a redox relay, capable of successfully regenerating dyes at only 0.1 eV overpotential. Strikingly, under 1000 lux indoor illumination their solar to electrical power conversion efficiency was found to be 28.9%, outperforming conventional silicon and even GaAs based photovoltaics in ambient conditions and thus paving the path to applications in IoT devices.^20,23^ To enable large area and sustainable production, the liquid electrolyte in DSCs needs to be replaced by a solid charge-transport material, however, current commonly used organic hole transport materials (such as spiro-MeOTAD) are limited in conductivity, stability and tunability.^24^ Contrarily, copper coordination complex-based hole transport materials (HTMs) demonstrated a new concept for solid-state DSCs (ssDSCs) with a stable and record-breaking solar cell efficiency of 11.7%.^25^

Considering the co-sensitization of dyes as a strategy to shape the TiO_2_/dye/electrolyte interface rather than the traditional approach of panchromatic extension of the spectral response,^26^ we designed DSCs that maintain a high photovoltage specifically under ambient light. Unfavourable electron back-transfers from the photoanode to the Cu^II/I^(tmby)_2_ electrolyte are supressed, and as a result we recorded a photovoltage of 910 mV, translating into a PCE of 34.0%, 32.7% and 31.4% under 1000, 500 and 200 lux of fluorescent light, respectively. Such photovoltaic conversion efficiencies deem DSCs the power sources of choice for IoT devices and wireless network sensors in ambient environments. IoT devices equipped with an array of these photovoltaic cells and a small energy buffer operate autonomously and therefore do not require long-term maintenance, such as battery replacements.^27^ Further, the use of light-driven, autonomous devices leads to a paradigm shift of energy usage: unlike battery-supported systems, which contribute to 10 billion dry-cell batteries produced annually, all surrounding energy can be harvested and used to the maximum of its availability.^28^

**Fig. 1:**
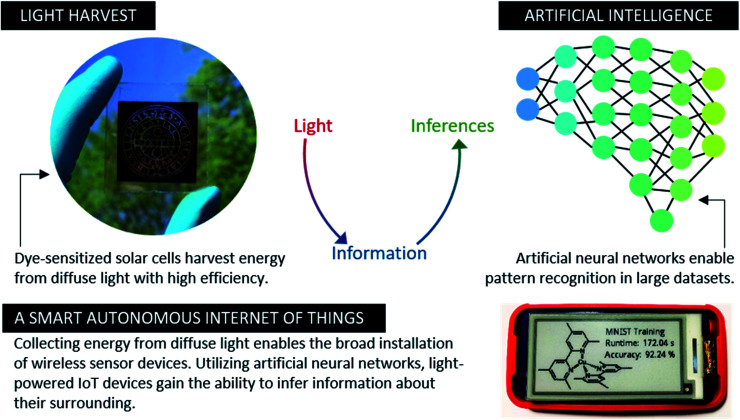
Fully autonomous IoT devices powered by harvested ambient light directly convert photons into computational information.

Implementing artificial intelligence directly on-device benefits such IoT sensor networks to a large extent (Fig. 1). IoT devices with pre-trained artificial neural networks (ANN) can directly infer or classify information about their surroundings, rather than communicating information through wireless networks. Reduction of the overall communication in the network is beneficial especially upon execution of heavy computational tasks such as advanced image recognition.^29–32^ As an additional advantage, on-device machine learning enables IoT devices to adapt to changing environments. In particular, they can self-optimize their energy consumption, perform demanding computations when ambient light is strongest and adaptive sleep during other times.^33–35^ Therefore, the combination of machine learning, environmental sensing, and photovoltaic cells as power sources lead to beneficial synergies. While the concept of machine learning on autonomous light-powered IoT nodes has been discussed broadly,^27,36,37^ complete pilot implementations are yet to be reported.

We demonstrate that our photovoltaic cells provide sufficient power from ambient light to an IoT node capable of sensing and communicating data within a wireless network, even when experiencing longer periods of darkness and hence no available energy. Photovoltaic cells were then used as a power source to train an artificial neural network on an IoT device and to use said neural network to infer information. Such self-powered and smart IoT devices employing machine learning are set to define technology for the next decades – based on distributed energy harvesters as power sources.

## Experimental

### Materials

Unless otherwise noted, chemicals were purchased from Sigma-Aldrich (Stockholm, Sweden) and used without further purification. Dyes XY1 and L1 were received from Dyenamo AB (Stockholm, Sweden). Copper complexes bis-(4,4′,6,6′-tetramethyl-2,2′-bipyridine)copper^II/I^ bis(trifluoromethanesulfonyl)imide, (Cu^II/I^(tmby)_2_ TFSI_(2)_), were synthesized according to Ferdowsi *et al.* and Saygili *et al.*^38,39^ For the latter, Cu^II^TFSI_2_ was purchased from TCI Chemicals (Zwijndrecht, Belgium).

### Solar cell fabrication

Generally, the fabrication of solar cells followed procedures as described in our previous reports.^40,41^ On cleaned (RBS solution, water, ethanol, UV-ozone) Nippon sheet glass (Pilkington, St. Helens, UK), 10 Ω sheet resistance, a dense TiO_2_ layer was deposited *via* spray pyrolysis at 450 °C from a 0.2 M titanium tetraisopropoxide, 2 M acetylacetone solution in isopropanol. Subsequently, 0.25 cm^2^ (0.5 cm × 0.5 cm), 3.2 cm^2^ (4 cm × 0.8 cm) or 8 cm^2^ (8 cm × 1 cm) TiO_2_ photoanodes were screen-printed (Seritec Services SA, Corseaux, Switzerland) from DSL 30 NRD-T (Dyesol/GreatCellSolar, Queanbeyan, Australia) colloidal (30 nm) TiO_2_ paste (4 μm). After brief drying at 120 °C, a scattering layer (Dyesol/GreatCellSolar WER2-0, 400 nm) was screen-printed onto of the mesoporous film (4 μm), followed by gradual heating towards a 30 minute sintering step at 450 °C. The substrates were post-treated with a 13 mM aqueous TiCl_4_ solution for 30 min at 70 °C and then sintered again at 450 °C for 30 min. After cooling, titania films were immersed into dye solutions for 16 h, which were prepared as reported in literature:^42,43^ 0.1 mM XY1 with 1 mM chenodeoxycholic acid in chloroform/ethanol 3 : 7 (similar for XY1b); 0.5 mM L1 in acetonitrile; 0.1 mM D35 in acetonitrile : *tert*-butanol; 0.1 mM Y123 with 1 mM chenodeoxycholic acid in acetonitrile : *tert*-butanol. The sensitizer solutions for XY1:D35 and XY1b:Y123 were mixed according to literature procedures.^20,21^ The mixing ratios for the sensitizer solutions of XY1 : L1 were studied according to Table S2.† PEDOT counter electrodes were manufactured *via* electro-polymerization of 3,4-ethylenedioxythiophene from a 0.01 mM aqueous solution with 0.1 M sodium dodecyl sulphate as previously studied in our laboratory.^44^ The redox electrolyte solutions for liquid DSCs were prepared with 0.2 M Cu(tmby)_2_TFSI and 0.04 M Cu(tmby)_2_TFSI_2_, 0.1 M lithium bis(trifluoromethanesulfonyl)imide and 0.6 M 4-*tert*-butylpyridine in acetonitrile. For photovoltaic cells powering IoT devices, propionitrile served as electrolyte solvent. Cells were assembled using ThreeBond (Dusseldorf, Germany) 3035B UV glue and cured with a CS2010 UV-source (Thorlabs, Newton, NJ, USA). The electrolyte was vacuum-injected through a hole in the counter electrode which was then sealed with a thermoplastic film and a glass cover slip. Solid-state DSCs were generally fabricated in a similar ‘sandwich’ layout. After electrolyte injection, cells were left to dry in ambient atmosphere for 72–96 hours. Devices were then sealed as described above before characterization.

### Solar cell characterization

Current–voltage measurements were carried out in ambient air under AM 1.5G illumination using a self-calibrating Sinus-70 solar simulator (Wavelabs, Leipzig, Germany). An X200 source meter (Ossila, Sheffield, UK) was used to assess solar cell performance (scan speed 100 mV s^−1^). A mask was employed to confine the active solar cell area to 0.16 cm^2^. Ambient light characterization was carried out with a Warm White 930 18 W fluorescent tube (OSRAM, Munich, Germany), and a PGSTAT 100 potentiostat (Metrohm Autolab, Utrecht, The Netherlands) was utilized to record the current–voltage characteristics. The lamp spectrum is illustrated in the ESI, Fig. S6.† The stabilized illumination intensity was calibrated with a commercial lux meter (Clas Ohlson, Insjön, Sweden) before measurements. Values of illumination intensity were cross-checked with lux meters from different manufacturers. The entire active photovoltaic area of the devices was used during indoor characterization to mimic diffuse light conditions.

### Incident photon-to-current conversion efficiency (IPCE)

IPCE spectra were recorded with an ASB-XE-175 xenon light source (10 mW cm^−2^) (Spectral Products, Putnam, CT, USA) and a CM110 monochromator (Spectral Products, Putnam, CT, USA). The photocurrent was measured with a U6 digital acquisition board (LabJack, Lakewood, CO, USA). The setup was calibrated with a certified silicon reference cell (Fraunhofer ISE, Munich, Germany). Photocurrents were integrated based on the spectral distribution of sunlight (AM 1.5G).^45^

### Electron lifetime measurements

Electron lifetimes were investigated with a 1 W white LED (Luxeon Star, Lethbridge, Canada). Kinetics in the solar cell were probed by applying square-wave modulations to the light intensity. The solar cell response was tracked by a digital acquisition board (National Instruments, Austin, Texas) and fitted with first-order kinetic models.

### Photoinduced absorption spectroscopy (PIA)

PIA spectra were recorded using square-wave-modulated blue light (1 W, 460 nm) (Luxeon Star, Lethbridge, Canada) for excitation, while as a probe a white light (20 W tungsten-halogen) was used, which was focused on a SP-150 monochromator (Acton Research Corp., Birmingham, AL, USA) with a UV-enhanced Si-photodiode. At sample location, pump and probe light intensities were estimated about 80 W m^−2^ and 100 W m^−2^, respectively. The sample response was assessed with a SR570 current amplifier and a SR830 lock-in amplifier (Stanford Research Systems, Reamwood, CA, USA).

### Transient absorption spectroscopy (TAS)

TAS spectra were recorded using a frequency-tripled Q-switched Nd-YAG laser as pump and a xenon arc lamp (continuous wave) as probe light source. The laser system was set to 520 nm excitation wavelength with a S12 Quanta-Ray optical parametric oscillator (Spectra Physics, Santa Clara, CA, USA) to provide 1 mJ, 13 ns pulses at an operating frequency of 10 Hz. The sample was positioned at an angle of 45° between the light sources, yielding a 0.35 cm^2^ cross-sectional active area. The sample response was analysed with an L920 detection unit (Edinburgh Instruments, Livingston, United Kingdom) containing a monochromator, an R928 photomultiplier and a TDS 3052B oscilloscope (Tektronix, Beaverton, OR, US).

### Raman spectroscopy

Raman spectra were collected using an InVia Renishaw Raman in confocal mode using a 50× objective, a frequency doubled Nd:YAG laser operating at 532 nm, and a Rayleigh line filter cutting 80 cm^−1^ into the Stokes part of the spectra. A 2400 lines mm^−1^ grating was used and the 520.5 cm^−1^ line from Si was used as a calibration giving a resolution of 1 cm^−1^. Raman spectra were recorded at different spots to confirm material homogeneity. Similar results were obtained upon repetition of the measurements with varying laser intensities.

### MNIST training

A Raspberry Pi Zero was used to benchmark the training of the machine learning. The training script was executed automatically after booting the system and results were displayed on an e-ink display. Four DGHQ 5.5 V 5 F supercapacitors with a total capacitance of 20 F were charged and the Raspberry Pi Zero was then powered from the supercapacitors using a commercial DC–DC boost converter with a standard USB cable.

### Wireless sensor node

An energy harvesting circuit using a diode, a AVX 6.0 V 0.47 F supercapacitor and a physical switch was used to power an ATmega328P microcontroller within its operational voltage. Arduino was used as the development platform for availability of reference implementations and libraries. The microcontroller was programmed to run at 8 MHz to allow for low-voltage operation. An nRF24L01+ wireless transceiver was used due to its low power requirements. No voltage regulator was used to limit energy losses to the microcontroller and the leakage current of the used supercapacitor (Fig. S10A†). The wireless receiver side was running continuously, deserializing and logging the incoming data packages into a database for visualization and data analysis. On the transmitter side, adaptive sleep was implemented using a PID-loop, using the internal voltage of the microcontroller as a set point. 2500 mV was selected as a set point for the internal voltage. Values for the proportional (*K*_p_), integral (*K*_i_) and derivative (*K*_d_) terms were experimentally evaluated and were the same for all experiments (*K*_p_ = 1, *K*_i_ = 0.05, *K*_d_ = 0.05). Intermittent sleep intervals were multiples of 10 seconds, with a maximum of 240 sleep cycles (*i.e.* 40 minutes).

### Benchmarking structure

All benchmarks used the same code structure, where the current–voltage profile of the microcontroller is acquired to determine the number of its sleep cycles. The workload was always executed before serializing the data package and transmitting it to the receiver. The pseudocode running on the energy harvesting circuits is outlined in Fig. S10B.†

### Heartbeat benchmark

The heartbeat benchmark sent a wireless communication package to the receiver, followed by 250 ms of sleep to limit the number of communication packages.

### Dhrystone benchmark

The Dhrystone benchmark was based on version 2.1 of the original Dhrystone code, with further modifications to allow execution on the ATmega328P. The result of the Dhrystone benchmark was calculated based on the time the microcontroller was going to sleep, resulting in an average MIPS (million instructions per second) rating. While the workload was always executed at the configured speed of 8 MHz, longer sleep times lead to lower average performance values.

### MNIST inference benchmark

The MNIST inference used a pre-trained network to infer the result of a given image. The network was pre-trained on a PC and compiled into the source code. An MNIST image was requested during the startup of the wireless node. The computation of the network was executed 100 times to be able to calculate the average required energy and number of photons per inference.

### MNIST robustness

To verify the robustness of the neural network, a 16 × 10 sample of MNIST digits was printed on paper using a laser printer. Images of the printed MNIST digits were then manually acquired using a USB camera. A simple imaging pipeline was then applied to the images before sending the data to the Arduino for inference: color conversion from RGB to grayscale, resizing to 14 × 14 pixels using nearest neighbor interpolation as well as edge-filtering of high values using a manually determined threshold. 127 out of 160 images were predicted correctly, yielding an inference accuracy of 79.4%.

### Code availability

The reported accuracies of the machine learning algorithms can be reproduced using the source code available from GitHub: https://github.com/freitaglab/LightToInformation. Code^46^ and models^47^ have been published on Zenodo.

## Results & discussion

### Efficient power generation under ambient light

**Fig. 2:**
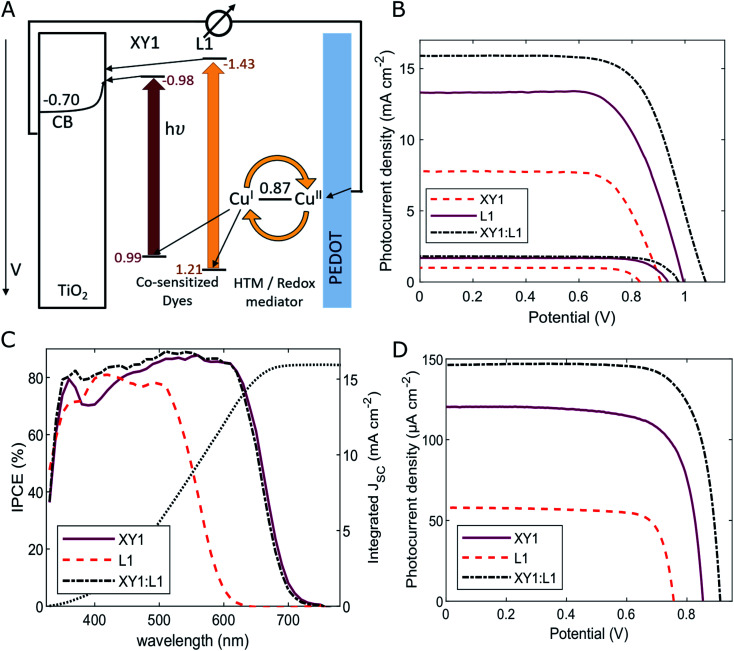
(A) Working principle of the XY1:L1-sensitized DSC. (B) Photovoltaic performance under simulated sunlight (AM 1.5G, 100 mW cm^−2^) and 10% sunlight, (C) incident photon to current conversion efficiency spectra (IPCE), and (D) photovoltaic performance under 1000 lux fluorescent light (303.1 μW cm^−2^) of XY1, L1 and XY1:L1 co-sensitized DSCs. Corresponding parameters are listed in Table 1.

Co-sensitization of dyes to extend the photoresponse of DSCs has been studied intensively throughout the literature (Fig. 2A). High solar cell performances are commonly achieved by a spectral combination of the broad photocurrent collection of small-transition-energy dyes with the large photovoltage generated by dyes with a large transition energy. Towards ambient light conversion, however, there are several other factors to consider. First, the spectral conversion response of the DSC should be judiciously tuned to the source of ambient lighting rather than aiming for a sole broadening of the absorption domain.^20^ Secondly, at low light intensities, the suppression of recombination processes plays a crucial role in the DSC performance. To avoid undesired back-transfers of electrons after their injection into the TiO_2_ conduction band, the adsorbed dyes need to protect the TiO_2_ surface from electronic interaction with the electrolyte.^48^ In particular, copper coordination complexes are known to show high recombination rates with electrons in the FTO and TiO_2_ in comparison to their cobalt-based counterparts.

Herein, we report on DSCs with Cu^II/I^(tmby)_2_ (tmby = 4,4′,6,6′-tetramethyl-2,2′-bipyridine) electrolyte based on a combination of sensitizers XY1 and L1 (Fig. S1†). Under simulated sunlight (AM 1.5G, 100 mW cm^−2^), the best device reached a photovoltage of 1080 mV, a photocurrent density of 15.9 mA cm^−2^, a fill factor of 0.67 and a power conversion efficiency (PCE) of 11.5% (Fig. 2B, Table S1†).

**Fig. 3:**
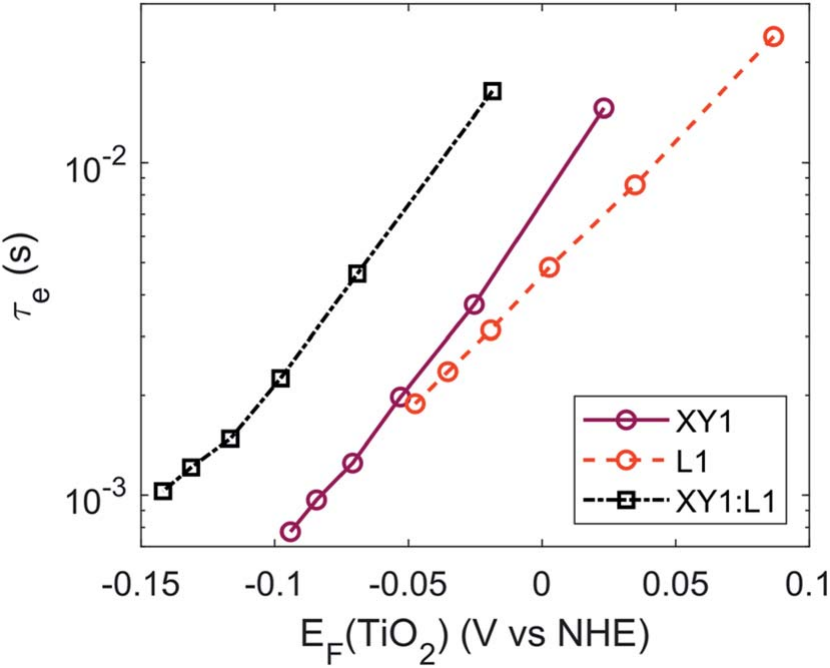
Electron lifetime in XY1, L1 and XY1:L1-sensitized solar cells.

Strikingly, the photovoltage of the co-sensitized DSCs largely exceeded the photovoltage generated by either dye alone. Devices based on sensitizer XY1 reached 1000 mV whereas the yellow L1 dye, despite the larger transition energy *E*_0–0_ of 2.64 eV, only generated a *V*_OC_ of 910 mV. In such case of a single sensitizer, the oxidized species of the redox mediator, here Cu^II^(tmby)_2_, can approach uncovered spots on the TiO_2_ and FTO surface and lead to electron recombination. The two sensitizers used in this study complement each other sterically in terms of TiO_2_ surface coverage due to the large difference in molecule size (Fig. S1†). The much smaller L1 dye molecules can occupy the surface area between larger XY1 molecules. As a result, a denser monolayer is formed, which passivates the surfaces of FTO and TiO_2_. Consequently, electron back-transfer from the TiO_2_ conduction band or FTO surface to the redox mediator is suppressed. Long electron lifetimes across the TiO_2_/XY1:L1/Cu^II/I^(tmby)_2_ interface further confirm supressed recombination in the co-sensitized DSCs (Fig. 3 and S4†).^48,49^

Due to the large electronic transition energy in the L1 dye, a larger quantity of high-energetic electrons is injected into the TiO_2_ conduction band, thus raising the TiO_2_ Fermi energy. As a consequence, the open-circuit voltage of the cell increased to 1080 mV. Lowering the illumination to 10% sunlight causes a small drop in the *V*_OC_ of XY1:L1-sensitized solar cells to 980 mV, while leading to an increase in PCE up to 13.7% (Fig. 2B and Table S1†).

Complementary light absorption of the two sensitizers XY1 and L1 allows for more effective photon collection and results in a greater number of electrons in the TiO_2_ conduction band. In the spectral region around 380–430 nm, DSCs employing a sole red sensitizer suffer from competitive light absorption by the orange Cu^II/I^(tmby)_2_ electrolyte, which infiltrates the mesoporous dye/TiO_2_ scaffold. The yellow dye L1 complements the absorption of the red/purple dye XY1 in the green-to-blue region around 400 nm. In this wavelength domain, incident-photon-to-current-conversion efficiency (IPCE) spectra of devices solely sensitized with the red dye XY1 indicate a reduced photocurrent collection (Fig. 2C). The L1 dye (*λ*_0–0_ of 404 nm) adds optical density around 400 nm and counters competitive light absorption by the Cu^II/I^(tmby)_2_ electrolyte. As a result, a larger number of photons is absorbed and DSCs with XY1:L1 as co-sensitizers exhibit a photon conversion efficiency above 80% over a broad spectral range from 350 to 630 nm. In addition, we found that both XY1 and L1 sensitizers are rapidly regenerated by the Cu^II/I^(tmby)_2_ electrolyte (Fig. S5†). In our study, the combination of XY1 and L1 dyes outperformed previously studied prominent co-sensitizers XY1:D35 (ref. 20) and XY1b:Y123 (ref. 21) (11.0% and 10.9% power conversion efficiency, respectively; Fig. S2, Table S1 and S3†).

Performance of photovoltaic devices was tested under ambient lighting with an OSRAM 930 18 W fluorescent tube. Due to close matching of the sensitizer composition to the lamp spectrum (Fig. S6 and Table S2†), XY1:L1 co-sensitized cells maintained a *V*_OC_ of 910 mV and collected 147 μA cm^−2^ of photocurrent density with a fill factor of 0.77 at 1000 lux of illumination (Fig. 2D, S2C, S3A,†Table 1, S3 and S4†). The cells generated 103.1 μW cm^−2^, corresponding to 34.0% power conversion efficiency, which, to the best of our knowledge, ranks amongst the highest in literature and atop DSC reports. The 97.0 μW cm^−2^ steady-state power output of the cells under load potential was identified to translate to 32.0% conversion efficiency (Fig. S11A†). At lower light intensities of 500 and 200 lux, the cells converted 49.5 and 19.0 μW cm^−2^ at 32.7% and 31.4% power conversion efficiency, respectively (Fig. S3B† and Table 1).

**Table 1:** Photovoltaic characterization of XY1-, L1-, and XY1:L1-sensitized DSCs under 1000, 500 and 200 lux (303.1, 151.5 and 60.6 μW cm^−2^) lux fluorescent light (normalized short-circuit current density and power output in parentheses)

	XY1 1000 lux	L1 1000 lux	XY1:L1 1000 lux	XY1:L1 500 lux	XY1:L1 200 lux
*V*_OC_ (mV)	850	750	910	880	840
*J*_SC_ (μA) (μA cm^−2^)	30.0 (120)	14.5 (58)	36.7 (147)	18.4 (73.4)	7.2 (29.0)
Fill factor	0.74	0.78	0.77	0.77	0.78
*P*_max_ (μW) (μW cm^−2^)	18.9 (75.4)	8.6 (34.4)	25.7 (103.1)	12.4 (49.5)	4.8 (19.0)
PCE (%)	24.9	11.3	**34.0**	**32.7**	**31.4**

Mathews *et al.* estimated that between 0.1 and 10 mW of power are required to operate common components of IoT devices, such as wireless data transfer.^14^ To provide such amount of power, efficient DSCs need to be manufactured beyond laboratory scale. As shown in Fig. S7A,† we assembled solar cells with active areas of 3.2 cm^2^ as well as 8 cm^2^. No significant performance drop was observed when characterizing larger cells under 1000 lux fluorescent light as the photovoltage remained above 900 mV even for 8 cm^2^ cells with only a slight decrease in photocurrent collection (Fig. S7B and Table S5†). The 3.2 cm^2^ cell reached a power output of 332 μW or 33.2%, while the 8 cm^2^ cell converted a total 740 μW at 30.6% power conversion efficiency.

**Fig. 4:**
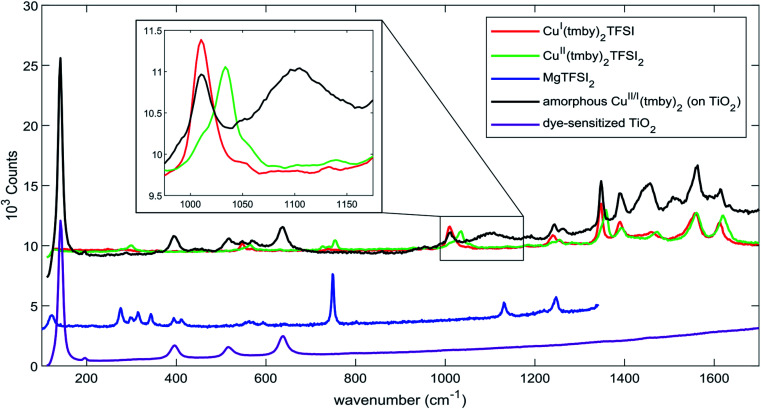
Raman spectra of Cu^II/I^(tmby)_2_ TFSI_(2)_ and MgTFSI_2_ (powders), DSCs sensitized with the XY1:L1 sensitizer combination as well as (sensitized) DSCs containing amorphous Cu^II/I^(tmby)_2_ TFSI_(2)_ hole transport material. The two latter spectra were recorded inside the ‘sandwich’ DSC. Spectra are offset for clarity.

The DSCs showed stable power outputs beyond evaporation of the electrolyte. As for the Cu^II/I^(tmby)_2_ electrolyte, its gradual drying in ambient atmosphere lead to the formation of a solid hole transporting material (Fig. S8†).^25,50–52^ We measured Raman spectroscopy directly inside the ‘sandwich’ solar cell to investigate the Cu^II/I^(tmby)_2_ hole transport material (Fig. 4).

A broad molecular vibration band, significant of the creation of an amorphous state, arises around 1100 cm^−1^, unknown to either Cu^II/I^(tmby)_2_, dye-sensitized TiO_2_ or TFSI counterions. Raman spectra further show a depletion of the Cu^II^ species in the solidified material and point towards accumulation of Cu^I^. Cao *et al.* indicated that decelerating the solidification of Cu^II/I^(tmby)_2_ suppresses the formation of grain boundaries, which in return increases the conductivity of the HTM due to less carrier trapping at such interfaces.^51^ Aydogdu *et al.* suggested a thermally-activated hole hopping mechanism as the transport mechanism in solidified copper coordination complexes,^53^ which in return suits the increase in photoconductivity with increasing photovoltage in solid-state DSCs as measured by Cao *et al.*

It is worth noting that, with respect to solar cells installed outdoors, indoor devices do not need to endure as harsh operating conditions concerning variations in temperature, humidity and level of irradiation. As a result, the expected lifetime of solar cells powering devices indoors increases greatly.^54,55^ Cao *et al.* demonstrated that solid-state ‘Zombie’ DSCs based on the Cu^II/I^(tmby)_2_ hole conductor show an increase in photovoltaic performance upon drying of the electrolyte; their devices maintained a power conversion efficiency above the initially recorded value after 40 days of unsealed ambient storage. Further, they noticed only a minor drop in power output after 200 hours of constant illumination.^51^ Zhang *et al.* further confirmed the durability of ‘Zombie’ solid-state DSCs during their 1000 hours stability testing.^25^ We monitored the evolution in device performance of XY1:L1-sensitized solar cells and found that, in agreement with previous reports, the formation of a solid-state hole conducting material leads to an increase in photocurrent, enhancing the total photoconversion efficiency of the cells under simulated sunlight (Fig. S9A and Table S6†).^50^ Partially inchoate penetration of the porous TiO_2_ layer by the amorphous Cu^II/I^(tmby)_2_ hole transport material leads to a slight drop in photovoltage. Nonetheless, devices maintained a power conversion efficiency of 30.0% under 1000 lux fluorescent light (Fig. S9B†) after evaporation of the electrolyte solvent, indicating high robustness for long time use, irrespective of sealing problems. In addition to evaluating the evolution of device performances, we carried out a twelve-day case study with our DSCs powering a wireless IoT device exposed to illumination and dark intervals. We observed no drop in the power supplied by the DSC array; the reader is here referred to the ensuing discussion of Fig. 6.

**Fig. 5:**
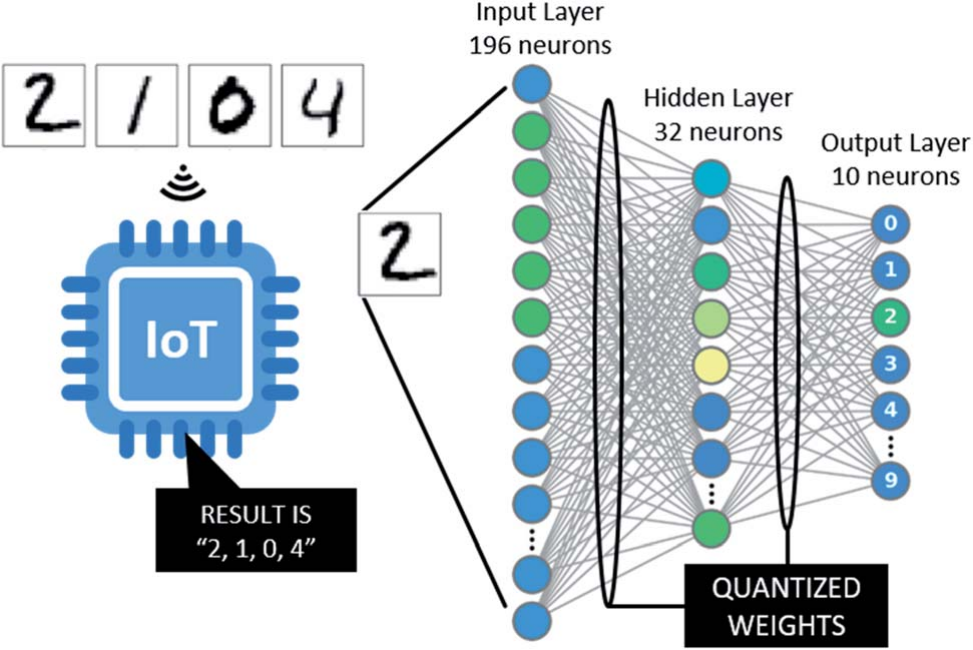
Structure of the neural network implemented on the self-powered IoT node.

**Fig. 6:**
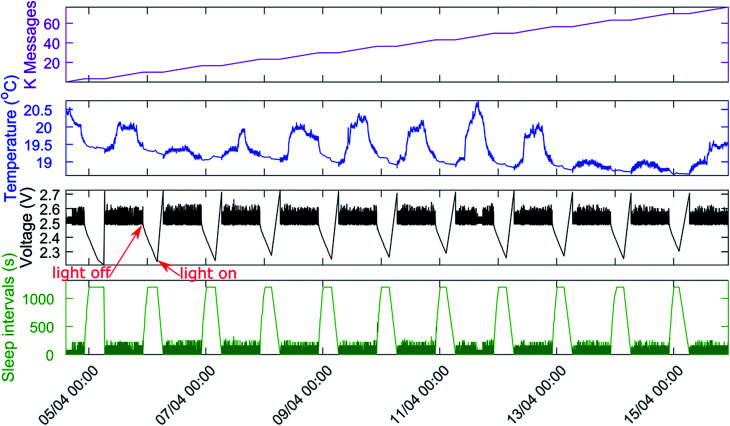
Atmega328P-based wireless node equipped with transceiver and AVX 6.0 V 0.47 F supercapacitor. The sensor is powered by five serial 3.2 cm^2^ photovoltaic cells (total 16.0 cm^2^). The sensor was tested in simulated light intervals of 16 hours “day” (1000 lux) and 8 hours darkness (10 pm–6 am, red arrows).

### Artificial intelligence on autonomous IoT devices

Artificial intelligence (AI) has found entry into many research fields from speech and image recognition, robotics, and autonomous cars to medical diagnosis, biology, and materials science.^56,57^ Machine learning (ML) was established in 1959 as a subfield of AI research.^58,59^ By utilizing methods from statistics and probability theory, the machine learns rules and strategies directly from data rather than having them imposed by a programmer. A substantial increase in available computational power accelerated the advance of ML in recent years. Large amounts of data can be easily collected and exchanged from many different sources over the internet.

The IoT makes use of this infrastructure and gathers data from a variety of low-cost sensing devices. Data processing and ML are usually executed on large servers, trying to achieve smart behaviour of the overall system. However, running a server for data acquisition and learning in many cases counteracts the energy savings achieved with said smart behaviour.^60^

Networks of IoT devices strongly benefit from the possibility to perform ML directly on the device. With a pre-trained model, the device can predict a global state solely from its locally gathered data and therefore reduce the need of communication within the network. Moreover, ML provides the possibility to predict quantities of interest by using only a small and easily accessible number of parameters. Directly accessing such quantities would require much more complicated systems, if they could be accessed at all. Furthermore, ML can help to reduce the number of devices needed to identify the global state of a system. Therefore, the combination of environmental sensing and inference through machine learning is ideally suited to adapt to the natural constraints of a fluctuating power source like the presented solar cell.

However, microcontrollers typically used in IoT nodes have very limited memory and processing power, constraining the possibility of training ML models directly on single IoT nodes. For an adaptive, self-learning IoT network it is thus necessary to provide a base station with sufficient computational power.

Here, we assessed the possibility to power both IoT nodes and a base station solely by harvested ambient light. An array of eight serial 8 cm^2^ photovoltaic cells (with a total of 64 cm^2^) illuminated with 1000 lux fluorescent light was used to power a Raspberry Pi Zero as a base station, using supercapacitors with a total capacitance of 20 F as an energy buffer. We used TensorFlow to design and train artificial neural networks.^61^ As a benchmark example, we implemented a neural network to categorize handwritten digits from the MNIST dataset.^62^ Image data was pre-processed and reduced in size to ensure that the trained network suits the limited memory capabilities of microcontrollers in the sensor network (*i.e.* an Atmega328P). The neural network consisted of an input layer with 196 neurons, a densely connected hidden layer with 32 neurons using a rectified linear unit activation and a densely connected layer with 10 neurons and softmax activation as output layer (Fig. 5).

One training epoch with 60 000 MNIST images and one verification run with 10 000 images resulted in an inference accuracy exceeding 90% (Table 2). The required 152 J were, in our example, charged within less than 24 hours at 1000 lux illumination, equalling 4.41 × 10^20^ photons or 7.32 × 10^−4^ einstein per training epoch.

**Table 2:** Investigated neural network (NN) structures with corresponding weight and bias count, size to store the network and achieved accuracy. The quantized two-layer NN was used on the IoT nodes. The maximum difference in accuracy between quantized NN and its full precision correspondence was 0.07% and was observed in both directions, better and worse than the full precision network

	Layers	Weights and biases	Computations per inference	Accuracy [%]
Deep six-layer NN by Cireşan *et al.*^64^	784–2500–2000–1500–1000–500–10	∼12 million (∼46 MB)	∼24 million	99.65
Large two-layer NN (15 epochs)	784–800–10	636 010 (∼2.5 MB)	1 270 400	98.3
Small two-layer NN (5 epochs)	784–64–10	50 890 (∼200 kB)	101 632	97
Two-layer NN on small images (5 epochs)	196–32–10	6634 (∼26 kB)	13 184	95.00 ± 0.17
Quantized two-layer NN on small images	196–32–10	6634 (∼6.5 kB)	13 184	94.99 ± 0.16

After training the neural network, the obtained weights and biases were post-processed and deployed to remote devices in the sensor network. Using 4 byte floating-point numbers for the 6634 weights resulted in 26 kB required memory to store the neural network. Converting floating-point numbers to 1 byte fixed-point numbers reduced the size by a factor of four, to the detriment of precision for each weight.^63^ Nonetheless, when evaluating the predictive power of the quantized network, the loss in accuracy was found no larger than 0.1% with respect to the predictive power of the full precision network (Table 2). The accuracy of the network using camera-acquired printed MNIST-digits was 80%. The quantization of neural networks is a crucial step to make ML inferences on low-power microcontrollers possible.

### Powering IoT nodes with dye-sensitized light harvesters

We consequently demonstrate a prototype of a fully self-powered intelligent IoT node inferring information based on a pre-trained artificial neural network, based on an ATmega328P microcontroller. Five serial 3.2 cm^2^ XY1:L1-sensitized cells (total 16 cm^2^) illuminated with 1000 lux fluorescent light provided the device with energy. We equipped the board with a AVX 6.0 V 0.47 F supercapacitor to serve as an energy buffer (Fig. S10†) and a rectifying diode to prevent capacitor discharging into the cells during dark intervals. Fig. S11B† shows an example of the charging curve of a serial array of photovoltaic cells (total 25.6 cm^2^) charging energy into a AVX 5.5 V 1.5 F supercapacitor. The microcontroller was configured at 8 MHz, which allows for an operating voltage of 1.8–5.5 V. Wireless communication was established through a low-power nRF24L01+ transceiver. For detailed information the reader is referred to Fig. S10.†

All benchmarks executed a workload inside a PID-control loop using the internal microcontroller voltage (which is equivalent to the capacitor voltage) as a set point, determining intermittent sleep intervals. In addition to MNIST machine learning inference, three benchmarks were executed as core workloads: heartbeat for continuous wireless communication, Dhrystone MIPS for assessment of computational performance assessment, and temperature sensing for day–night testing.^62,65^ All benchmarks were executed on fully untethered harvesters and results were wirelessly transmitted to a power-connected base station. The transmitted 12 byte serialized data package contained information about the package length, a package identifier, internal voltage, sensor data, message count, and the number of sleep cycles.

The heartbeat benchmark contained no sensor data and executed no further workload. Data was continuously sent at 282 ms per data package, of which 250 ms were an intentional sleep interval, giving an effective execution time of 32 ms. The internal voltage increased to an equilibrium between energy harvest and consumption, determined by the increased power consumption of the microcontroller at higher operating voltages, slower energy charging of the buffer and general leakage.

The Dhrystone MIPS benchmark was used in an adapted version in order to be executable on the microcontroller. The average VAX MIPS were calculated on-chip and included the calculated sleep time. An average computational performance of 0.413 VAX MIPS was recorded over a period of 24 hours. During that period, a total of 19 hours 52 minutes of sleep was protocolled, leading to an effective 4 hours 8 minutes or 17% active runtime of the microcontroller under full CPU-load.

Machine learning capabilities were benchmarked using a pre-trained two-layer network to categorize images of handwritten digits from the MNIST dataset, which were received wirelessly. Inferences were averaged over 100 computations before transmission. The computation of each inference consumed 0.947 mJ of energy in our pilot experiment, translating to 2.72 × 10^15^ photons or 4.51 × 10^−9^ einstein per inference and pose an important benchmark for future approaches. 16 cm^2^ of photovoltaic area provided sufficient energy for one inference within just 581 ms of 1000 lux illumination.

We extended the benchmark to a simulated day–night indoor environment with 16 hours of 1000 lux illumination and 8 hours of darkness for twelve days, measuring the temperature as workload (Fig. 6). As an initial observation, the operating voltage on the microcontroller exhibits the same pattern of voltage decay during dark periods and voltage rise under illumination for the duration of the entire experiment. As a result, we conclude that the DSCs provide a constant amount of energy and exhibit excellent stability under 1000 lux illumination. On average, the wireless sensor transmitted data every 16 seconds during illumination intervals, well-ranging within common battery-driven wireless sensors. During ‘night’ intervals, the microcontroller used the energy stored in the AVX 6.0 V 0.47 F supercapacitor to ensure data transmission to the base station in intervals of minutes. The microcontroller operated continuously without shutting down, thus removing the requirement to save data to non-volatile memory.

While 1000 lux was chosen as a standard premise for this feasibility study, in many cases indoor IoT devices will not be illuminated with more than 200-500 lux (Fig. S3B† and Table 1).^14,66^ Nonetheless, as demonstrated in this pilot example, the operation at an equilibrium between execution of computational load and intermittent sleep cycles ensures that the IoT device adapts to the available light energy. It is without question worth noting that, certainly at illumination intensities dropping below 200 lux, the array of photovoltaic cells will slow down certain core computational workloads such as the energy-consuming training of an artificial neural network. Nonetheless, the large photovoltage of 840 mV at 200 lux allows the photovoltaic cells to provide a voltage within the operating range of many microcontrollers even at such low light intensities. Meanwhile, the harvested photocurrents follow a linear dependency on the intensity of illumination. As a result, the photovoltaic cells will continue to steadily charge the energy buffer.

## Conclusions

As a communicating and autonomous system of interconnected devices, a self-powered IoT embodies technological sustainability. An enhanced exchange of light and information in intelligent wireless sensor networks looks poised to transform the implementation of IoT devices. Through elimination of recombinative electron transfers across the photoactive interface, dye-sensitized solar cells based on dyes XY1 and L1 maintain over 910 mV of photovoltage under 1000 lux of fluorescent light, translating into 34.0%, 32.7% and 31.4% conversion efficiency at 1000, 500 and 200 lux of fluorescent light. Employing small arrays of photovoltaic cells, core workloads of IoT devices such as sensing and wireless communication were benchmarked. The possibility of inferring information from learned models was tested through the implementation of an artificial neural network: In our experimental set up, 152 J or 4.41 × 10^20^ photons were required to train and verify an artificial neural network; 0.947 mJ or 2.72 × 10^15^ photons were subsequently required to compute one inference.

## Funding

This work was supported by the Royal Society University Fellowship (URF\R1\191286), Swedish Energy Agency (43294-1), Swedish Research Council (2018-04570), Göran Gustafsson Young Researcher Prize 2019 and Olle Engkvist Byggmästare Stiftelsen (184-482). A. G. and M. R. acknowledge the Nanosystems Initiative Munich (NIM) by the German Research Foundation (DFG) for funding. H. M. acknowledges support by the EIT InnoEnergy PhD programme.

## Author contributions

M. F. conceptualized and directed the research together with A. G.; H. M. manufactured and characterized the solar cells; M. R. and R. F. prepared the program code; T. E. recorded the Raman spectra; R. S. contributed to analysis on MNIST image classification; R. F., H. M. and I. B. built the testing devices; H. M. wrote the manuscript and all authors contributed to compiling the manuscript.

## Conflicts of interest

Authors declare no competing interests.

## Supplementary Material

SC-011-C9SC06145B-s001

SC-011-C9SC06145B-s002
