# Supplementary material for: Dye-sensitized solar cells under ambient light powering machine learning: towards autonomous smart sensors for the internet of things
Source: Chem Sci. 2020 Feb 13;11(11):2895–906. doi: 10.1039/c9sc06145b (PMC8157489; doi:10.1039/c9sc06145b)
Supplement: SC-011-C9SC06145B-s002 [file SC-011-C9SC06145B-s002.pdf]

Electronic Supplementary Material (ESI) for Chemical Science.
This journal is © The Royal Society of Chemistry 2020

Supplementary Information for

Dye-sensitized solar cells under Ambient Light Powering Machine Learning: Towards autonomous smart sensors for Internet of Things

Hannes Michaels, Michael Rinderle, Richard Freitag, Iacopo Benesperi, Tomas Edvinsson, Richard Socher, Alessio Gagliardi, Marina Freitag

Correspondence to: marina.freitag@newcastle.ac.uk

This PDF file includes:

Figures S1-S11

Tables S1-S6

Fig. S1 Structures of dyes and copper complexes.

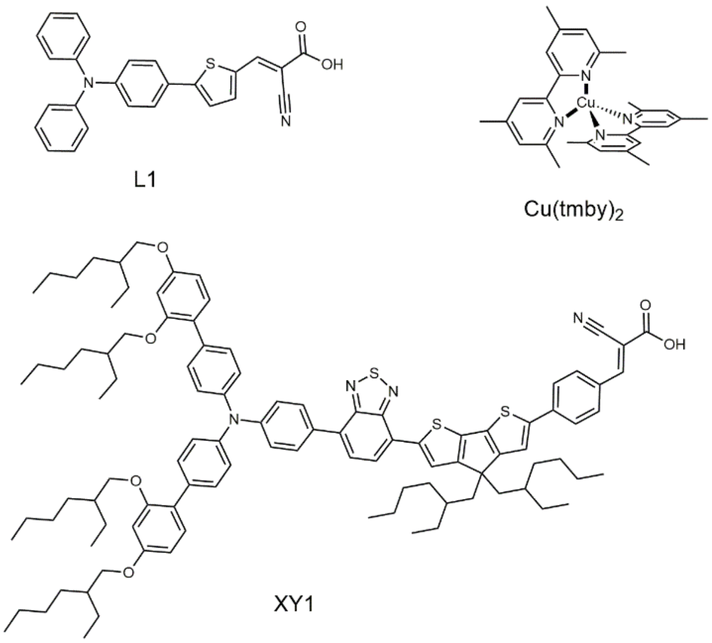

Fig. S1. Chemical structures of investigated dyes L1, XY1 as well as copper redox mediator $Cu(tmby)_2$ (counterions omitted for clarity).

Fig. S2. Photovoltaic characterization.

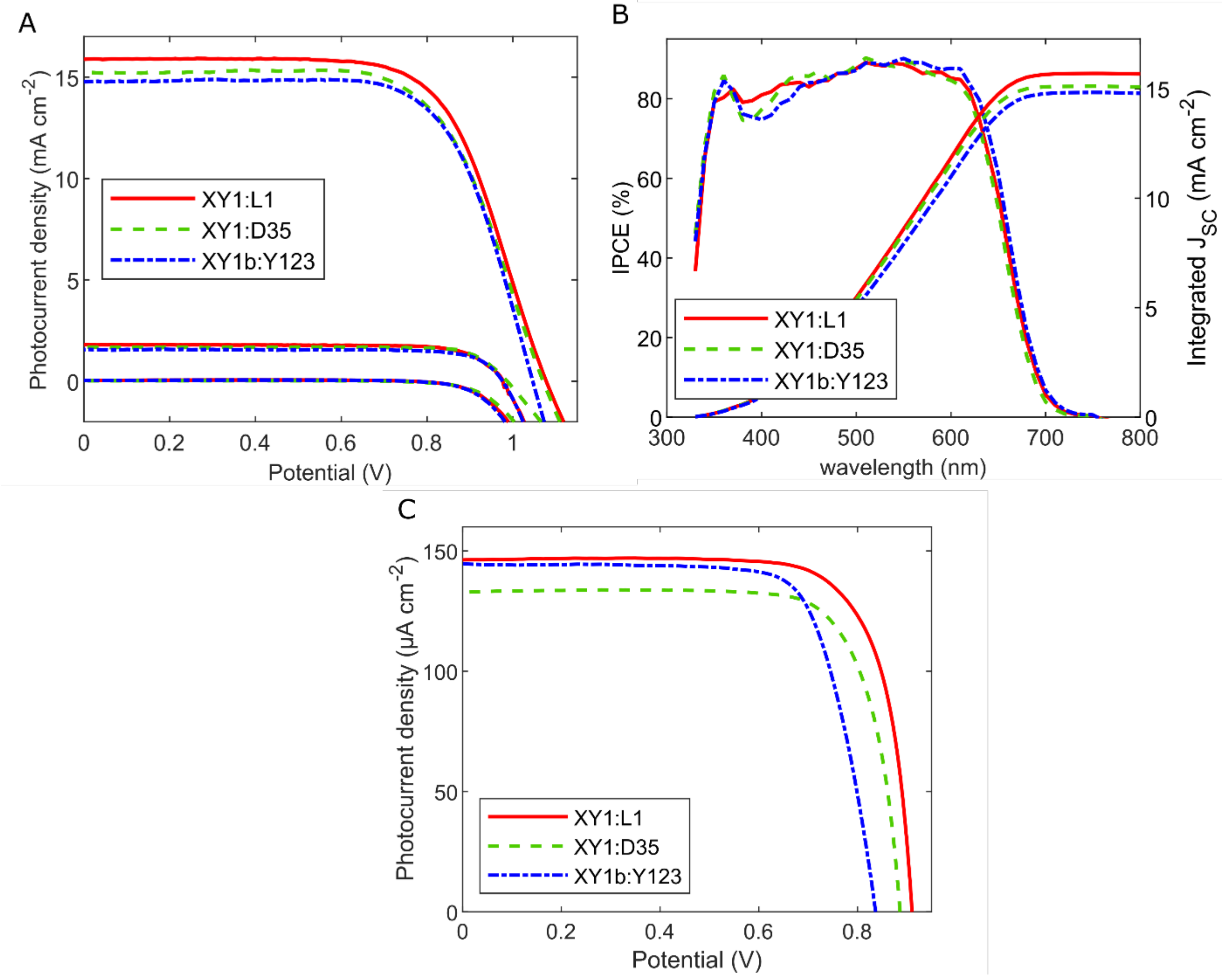

Fig. S2. (A) Photovoltaic performance under simulated sunlight (AM 1.5G, 100 mW cm^{-2}), 10% sunlight as well as dark currents and **(B)** Incident-photon-to-current-conversion efficiency comparing XY1:L1-sensitized solar cells to sensitizer combinations XY1:D35 and XY1b:Y123. All corresponding parameters are listed in Table S1. **(C)** Photovoltaic performance of XY1:L1, XY1:D35 and XY1b:Y123 sensitized solar cells illuminated with 1000 lux fluorescent light. Corresponding parameters are listed in Table S3.

Fig. S3. Photovoltaic characterization.

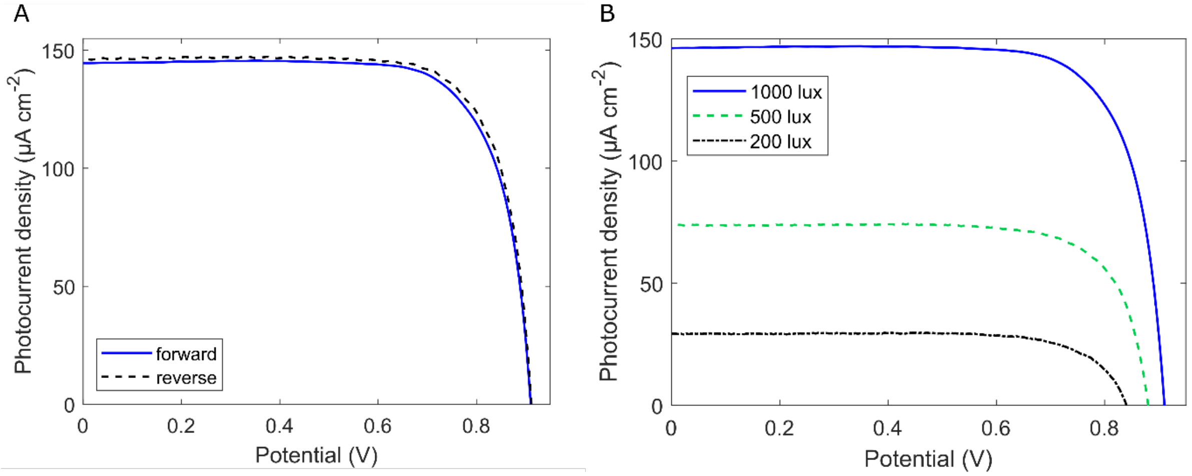

Fig. S3. (A) Forward (33.1%) and reverse (34.0%) scan of an XY1:L1-sensitized solar cell under 1000 lux fluorescent light. Full parameter list in Table S4. **(B)** Photovoltaic performance of an XY1:L1-sensitized solar cell illuminated by a fluorescent lamp at different intensities. Parameters in Table 1.

Fig. S4. Electron lifetime and transport time.

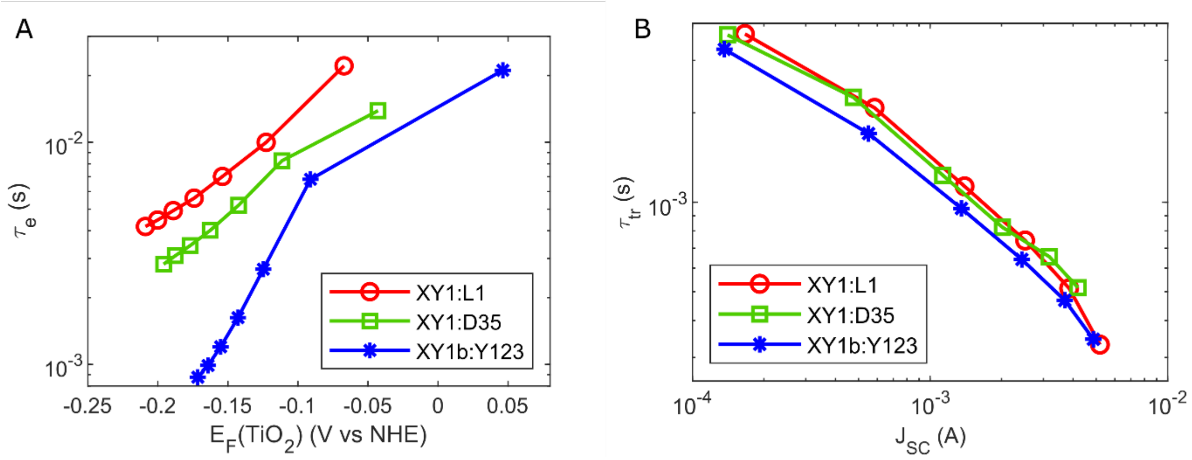

Fig. S4. **(A)** Electron lifetime in XY1:L1, XY1:D35 and XY1:Y123-sensitized solar cells. The measurements were aligned to the effective Fermi energy of electrons in the TiO_2 (in Volts vs NHE), which was calculated via $E_F = V_{OC} – E_{redox}$, where V_{OC} is the open-circuit voltage of the cell and Eredox represents the redox potential of the $Cu^{II/I}(tmby)_2$ redox electrolyte. **(B)** Transport time.

Fig. S5. Dye regeneration.

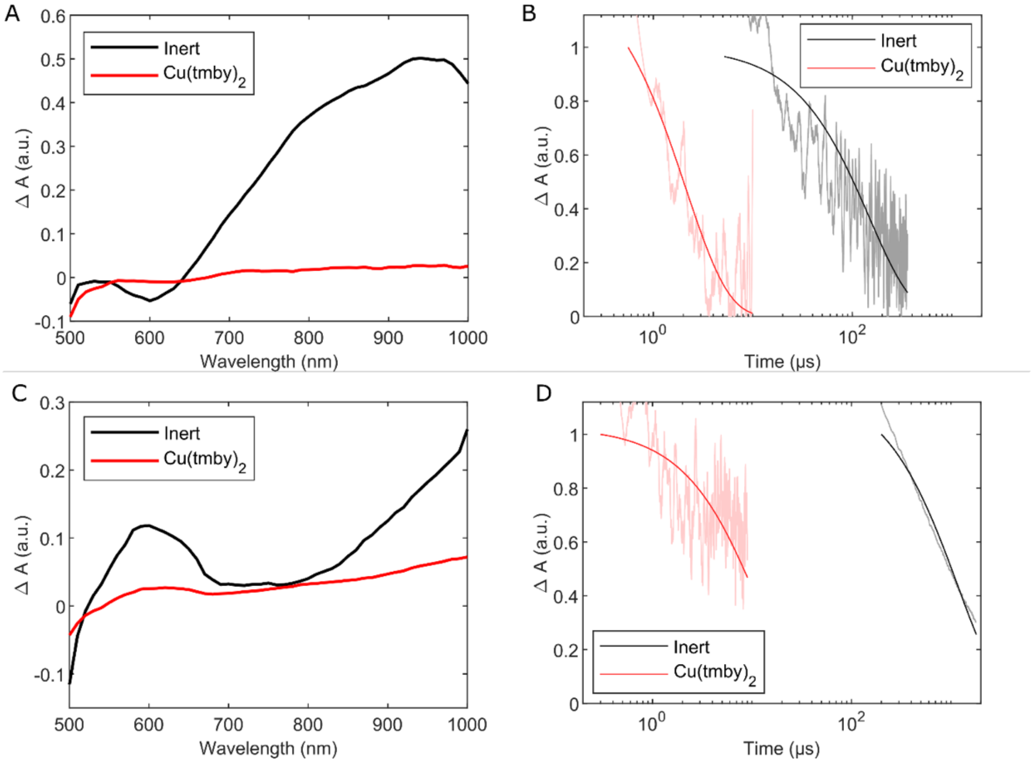

Fig. S5: (A) Photoinduced absorption spectra of XY1-sensitized photoanodes in inert and $Cu^{II/I}(tmby)_2$ electrolyte. **(B)** Transient absorption spectra of XY1-sensitized photoanodes in inert and $Cu^{II/I}(tmby)_2$ electrolyte with single-exponential fits. The pump wavelength was 555 nm, the absorption was monitored at 780 nm. Half-times of the absorption decay yield a recombination time of 33 μs in the inert measurement and a 1.9 μs regeneration time for the $Cu^{II/I}(tmby)_2$ redox couple, resulting in a regeneration efficiency of 94%. **(C)** Photoinduced absorption spectra of L1-sensitized photoanodes in inert and $Cu^{II/I}(tmby)_2$ electrolyte. **(D)** Transient absorption spectra of L1-sensitized photoanodes in inert and $Cu^{II/I}(tmby)_2$ electrolyte with single-exponential fits. The pump wavelength was 470 nm, the absorption was monitored at 620 nm. Half-times of the absorption decay yield a recombination time of 1.02 ms in the inert measurement and a 0.8 μs regeneration time for the $Cu^{II/I}(tmby)_2$ redox couple, resulting in a regeneration efficiency of 99.9%.

Fig. S6. Spectrum of the OSRAM fluorescent tube.

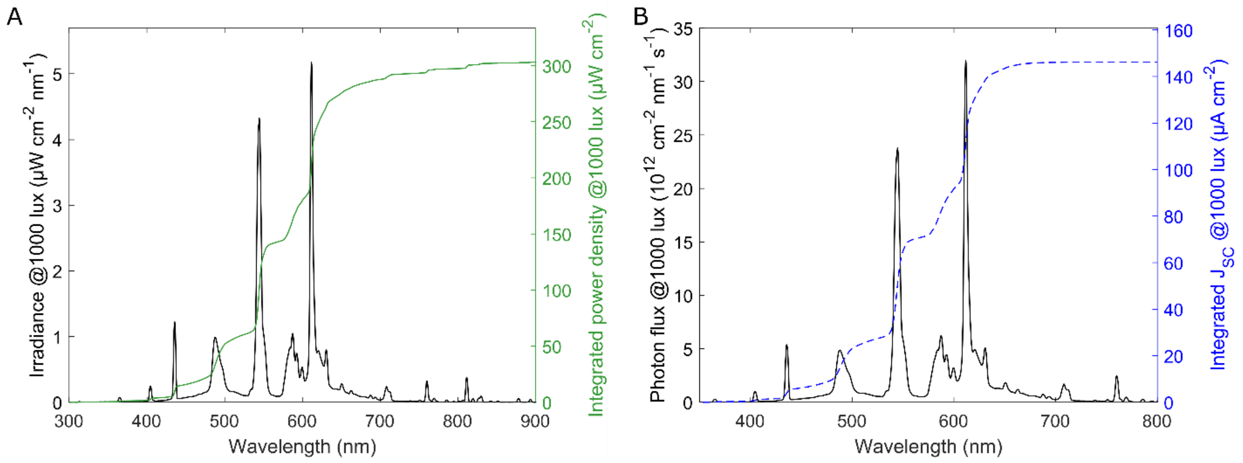

Fig. S6: (A) Spectrum of the Osram 930 warm white fluorescent tube. After calibration with a lux meter, the spectral distribution was weighed with the luminous efficiency function to obtain the power distribution (right axis). The power density was integrated to yield 303.1 µW cm^{-2}. **(B)** The photon flux of the fluorescent lamp integrated with the IPCE spectrum of an XY1:L1-sensitized solar cell (shown in Fig. 2C) to obtain a photocurrent density of 146 µA cm^{-2} (compare Fig. S2, Table S3: 147 µA cm^{-2}).

Fig. S7. Characterization of large DSCs.

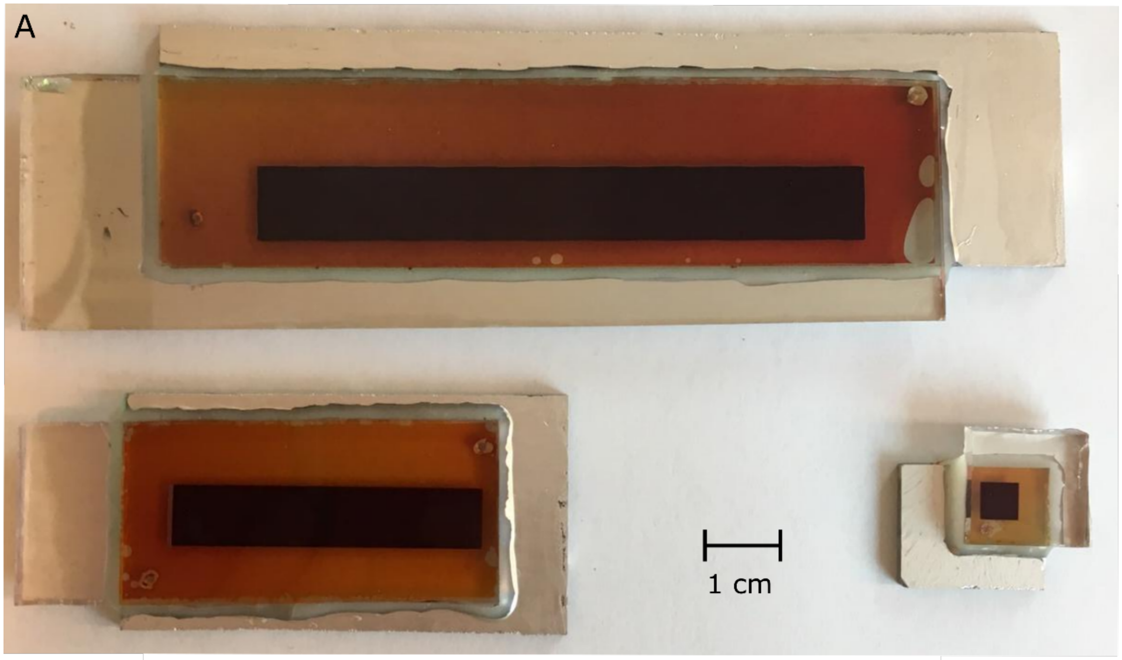

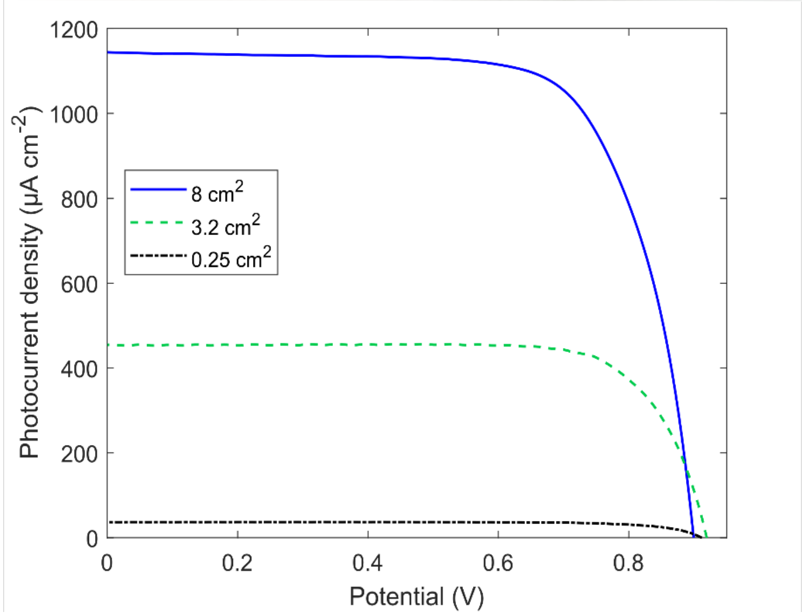

Fig S7: (A) 0.25 cm^2, 3.2 cm^2 (4 cm x 0.8 cm) and 8 cm^2 (8 cm x 1 cm) XY1:L1 sensitized solar cells with $Cu^{II/I}$(tmby)$_2$ electrolyte. **(B)** Photovoltaic characterization of XY1:L1-sensitized solar cells under 1000 lux fluorescent light. Corresponding parameters in Table S5.

s

Fig. S8. Fabrication of solid-state 'Zombie' DSCs.

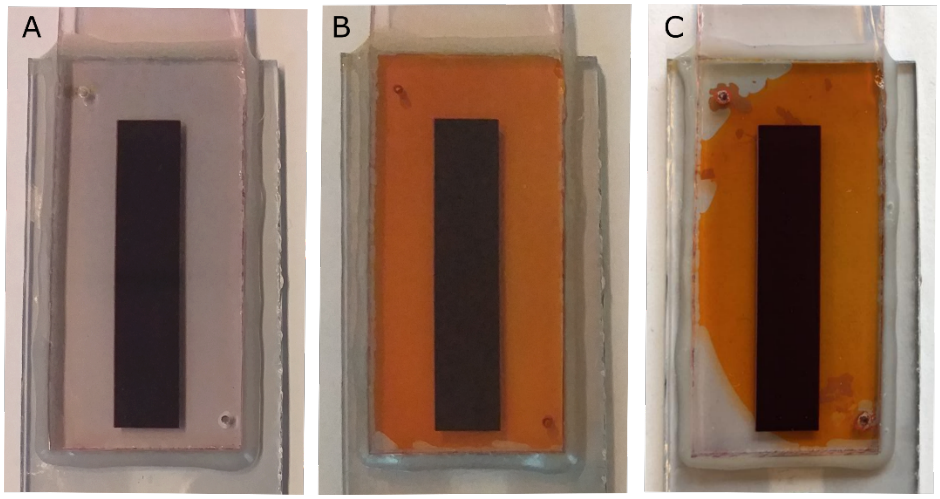

Fig. S8. The fabrication of solid-state 'Zombie' DSCs. **(A)** The XY1:L1 co-sensitized photoanode assembled with PEDOT counter electrode in inert electrolyte (0.1 M LiTFSI, 0.6 M *tert*-buylpyridine in acetonitrile). **(B)** Assembled cell filled with $Cu^{II/I}(tmby)_2$ electrolyte. **(C)** The electrolyte dries in ambient air to form the 'Zombie' solid-state DSC.

Fig. S9. Characterization of 'Zombie' solid-state DSCs.

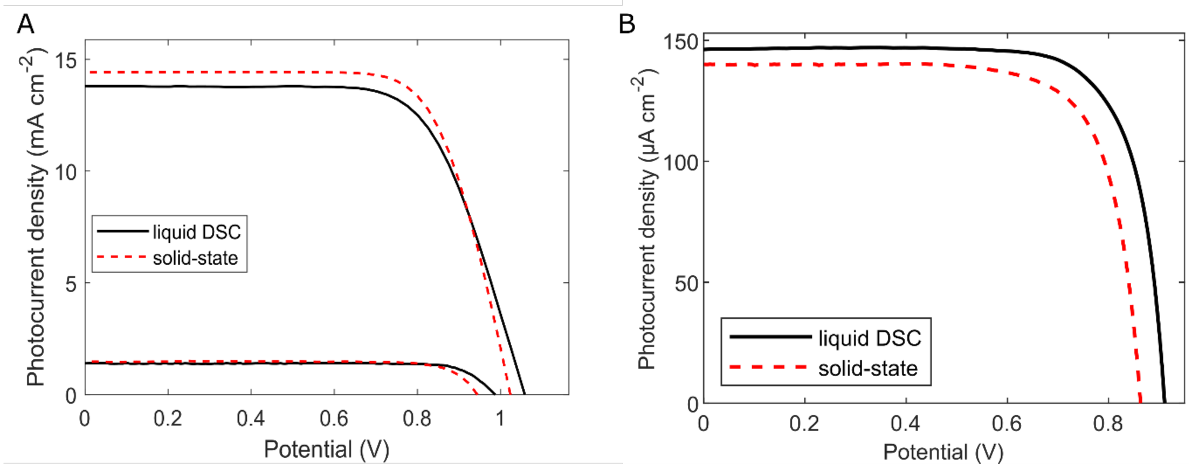

Fig. S9. (A) Photovoltaic characterization of liquid-DSCs and solid-state 'Zombie' DSCs with XY1:L1 as sensitizers and $Cu^{II/I}(tmby)_2$ as electrolyte / hole transport material under AM 1.5G simulated sunlight as well as 10% sunlight and **(B)** under 1000 lux fluorescent light. All corresponding parameters can be found in Table S6.

Fig. S10. Energy harvester and pseudocode.

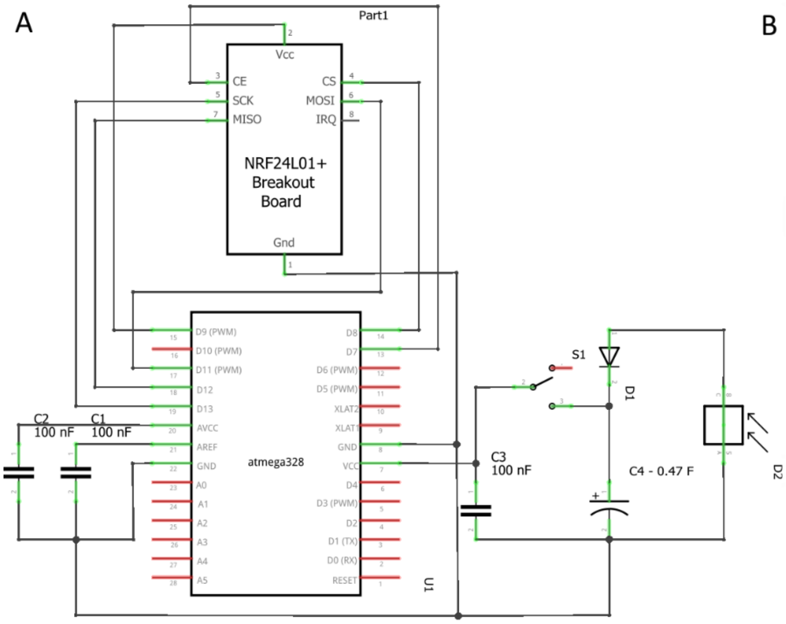

B

```
void function setup{
    initialize wireless communication
    initialize PID terms
}

void function loop{
    get vcc

    if (vcc > 2200) {
        PID compute sleep_loops

        execute workload(process, sense)

        serialize data package
        power up wireless communication
        transmit data package
        power down wireless communication

        for (i = 0; i < sleep_loops; i++) {
            sleep deeply 10 seconds
        }
    }
    if (vcc < 2200) {
        power up wireless communication
        transmit low power data package
        power down wireless communication
        for (i = 0; i < 12; i++) {
            sleep deeply 10 seconds
        }
    }
}
```

Fig. S10. (A) Schematic of the basic energy harvester with microcontroller U1, wireless transceiver P1, light harvester L1 and energy buffer (C4) **(B)** Pseudocode of benchmarks running on energy harvesting circuit

Fig. S11. Stabilized power output and charging of supercapacitor.

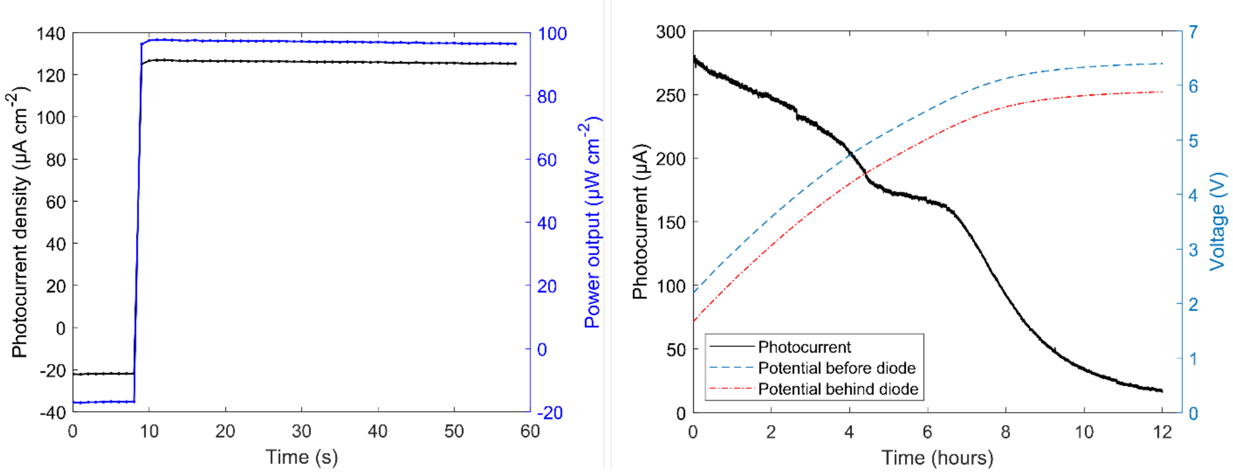

Fig. S11. (A) Steady-state power output at the maximum power point (MPP) of a DSC under 1000 lux fluorescent light, recorded at V_{MPP} = 0.77 V. A J_{MPP} of 126 µA cm^{-2} and P_{MPP} of 97.0 µW cm^{-2} translate into 32.0% steady-state power conversion efficiency. **(B)** A 1.5 F @ 5 V supercapacitor is being charged by an array of eight serial 3.2 cm-2 XY1:L1-sensitized solar cells through a rectifying diode. As the potential in the capacitor is building up (right), the photocurrent decreases. The *S*-shape of the charging curve is likely attributed to slight MPP mismatching within the serial solar cell array.

Table S1. Photovoltaic characterization of DSCs under simulated sunlight.

Table S1. Photovoltaic parameters for XY1:L1, XY1:D35 and XY1b:Y123 sensitized solar cells under AM 1.5G illumination (100 mW cm^{-2}). Photovoltaic parameters under 10% sunlight in parentheses. Scanning the champion XY1:L1-sensitized cell in darkness yielded a saturation current of 5 nA cm^{-2} as well as an ideality factor of 1.92.

	XY1	L1	XY1:L1	XY1:D35	XY1b:Y123
V_{OC} (mV)	1000<br>(930)	910<br>(830)	1080<br>(980)	1070<br>(980)	1050<br>(970)
J_{SC}<br>(mA cm^{-2})	13.3<br>(1.59)	9.4<br>(1.00)	15.9<br>(1.80)	15.3<br>(1.69)	14.7<br>(1.56)
$J_{SC,IPCE}$<br>(mA cm^{-2})	13.3	9.0	15.7	15.1	14.8
Fill Factor	0.67<br>(0.80)	0.71<br>(0.80)	0.67<br>(0.77)	0.67<br>(0.77)	0.70<br>(0.80)
PCE (%)	8.9<br>(11.8)	6.1<br>(6.7)	11.5<br>(13.7)	11.0<br>(13.0)	10.9<br>(12.1)

Table S2. Photovoltaic characterization of DSCs under ambient light: Sensitizer ratio.

Table S2. Photovoltaic characterization of XY1:L1 co-sensitized solar cells under 1000 lux fluorescent light. The first column indicates the relative molar ratios of sensitizers XY1 and L1 in the dye solution.

	V_{OC} (mV)	J_{SC} (μA cm^{-2})	Fill Factor	PCE (%)
XY1	850	114.2	0.78	24.6
5:1	850	120	0.76	25.5
2.5:1	870	138	0.77	30.5
1:1	890	143	0.77	32.3
**1:2.5**	**910**	**147**	**0.77**	**34.0**
1:5	910	140	0.76	31.9
L1	760	58	0.78	11.2

Table S3. Photovoltaic characterization of DSCs under ambient light: Sensitizer combinations.

Table S3: Photovoltaic parameters for XY1:L1, XY1:D35 and XY1:Y123-sensitized solar cells under 1000 (303.1 μW cm^{-2}) lux fluorescent light (normalized short-circuit current density and power output in parentheses).

	XY1	L1	XY1:L1	XY1:D35	XY1b:Y123
V_{OC} (mV)	850	750	910	880	840
J_{SC} (μA) ((μA cm^{-2}))	30.0 (120)	14.5 (58)	36.7 (147)	33.0 (132)	36.3 (145)
$J_{SC,IPCE}$ (μA cm^{-2})			146		
Fill Factor	0.74	0.78	0.77	0.77	0.75
P_{max} (μW) ((μW cm^{-2}))	18.9 (75.4)	8.6 (34.4)	25.7 (103.1)	22.4 (89.4)	22.7 (91.2)
PCE (%)	24.9	11.3	34.0	29.5	30.1

Table S4. Photovoltaic characterization of DSCs under ambient light: Hysteresis data.

Table S4: Hysteresis data for an XY1:L1-sensitized solar cell under 1000 lux fluorescent light (normalized short-circuit current density and power output in parentheses).

	forward	reverse
V_{OC} (mV)	910	910
J_{SC} (μA)<br>((μA cm^{-2}))	36.3<br>(145)	36.7<br>(147)
Fill Factor	0.76	0.77
P_{max} (μW)<br>((μW cm^{-2}))	25.7<br>(103.1)	25.1<br>(100.3)
PCE (%)	34.0	33.1

Table S5. Characterization of large DSCs under ambient light.

Table S5. Photovoltaic parameters for XY1:L1-sensitized solar cells under 1000 lux, fluorescent light (normalized short-circuit current density and power output in parentheses).

	0.25 cm^2	3.2 cm^2	8 cm^2
V_{OC} (mV)	910	910	900
J_{SC} (μA)<br>(($\mu A\ cm^{-2}$))	36.7<br>(147)	454<br>(142)	1140<br>(142)
Fill Factor	0.77	0.78	0.73
P_{max} (μW)<br>(($\mu W\ cm^{-2}$))	25.7<br>(103.1)	332<br>(100.3)	740<br>(92.5)
PCE (%)	34.0	33.2	30.6

Table S6. Statisctics of photovoltaic parameters.

Table S6. Photovoltaic parameters of liquid-electrolyte DSCs and solid-state ‘Zombie’ DSCs with XY1:L1 as sensitizers and $Cu^{II/I}(tmby)_2$ as electrolyte / hole transport material under AM 1.5G as well as 10% sunlight and under 1000 lux fluorescent light.
*Champion device.
‡Average of three batches of liquid-electrolyte DSCs (total of 40 cells).
†Average of those cells that were dried to characterize ‘Zombie’ ssDSCs (8 cells).

		V_{OC} (mV)	J_{SC} (mA cm^{-2}))	Fill Factor	PCE [%]
AM 1.5G simulated sunlight	DSC*<br>DSC‡<br>DSC†	1080<br>1060<br>1050	15.9<br>15.2<br>13.9	0.67<br>0.68<br>0.70	11.5<br>11.0<br>10.2
	ssDSC*<br>ssDSC†	1020<br>1020	14.5<br>14.3	0.72<br>0.71	10.7<br>10.4
10% sunlight	DSC*	980	1.80	0.78	13.7
	ssDSC*	940	1.47	0.80	11.2
1000 lux fluorescent	DSC*	910	0.147	0.77	34.0
	ssDSC*	860	0.137	0.77	30.0
